# Wideband parametric baseband macromodeling of linear and passive photonic circuits via complex vector fitting

**DOI:** 10.1038/s41598-023-41227-w

**Published:** 2023-09-16

**Authors:** Thijs Ullrick, Domenico Spina, Wim Bogaerts, Tom Dhaene

**Affiliations:** 1 IDLab, Department of Information Technology, Ghent University-imec, Ghent, Belgium; 2 Photonics Research Group, Department of Information Technology, Ghent University-imec, Ghent, Belgium; 3 The Center for Nano- and Biophotonics (NB-Photonics), Ghent, Belgium

**Keywords:** Mathematics and computing, Optics and photonics

## Abstract

A novel wideband parametric baseband macromodeling technique for passive photonic devices and circuits is presented. It allows to efficiently estimate the baseband scattering representations of a linear, passive photonic system as a function of a set of design variables, such as geometrical layout or substrate features. The proposed technique relies on the interpolation of macromodels computed via a complex vector fitting (CVF) algorithm, by adopting a methodology based on amplitude and frequency scaling that preserves, by construction, the physical properties of the system, such as causality, stability and passivity. For a specified combination of the design parameters, a rational CVF model is derived that can be simulated by a wide range of ordinary differential equation (ODE) solvers or circuit simulators. Additionally, time-domain simulations using the computed model can be performed at arbitrary optical carrier frequencies, thus allowing for the simulation of multi-wavelength systems. Two application examples are presented to demonstrate the flexibility and advantages of the proposed method.

## Introduction

Driven by datacom and telecom applications, and enabled by the growing number of mature manufacturing and prototyping facilities (fabs), there is a growing trend to scale the complexity of *photonic integrated circuits* (PICs)^1^. However, today there exists a wide gap between what the technology can deliver and the functionality engineers need to design and simulate PICs^2^. Since traditional electromagnetic (EM) modelling techniques^3–6^ are not suitable for directly simulating larger circuits, the more common approach is to use circuit-level modelling, which needs compact and efficient behavioral models that can substitute for the expensive EM simulations while ensuring a comparable accuracy.

In general, photonic components for PICs can be divided in two broad classes: active and passive devices. A great number of models for active components, which are usually formulated in the time-domain, has been presented in the literature: examples include lasers^7–9^, modulators^10–12^, and photodetectors^13–15^. However, the vast majority of photonic components are linear and passive devices whose behaviour is best defined in the frequency-domain in terms of a scattering matrix. However, since simulation of active and passive devices is always performed in the time-domain^16,17^, there is also a need for accurate and efficient time-domain models of passive devices. As many passive devices in photonics circuits are constructed using parametric layout designs (i.e. the user can adjust dimensions using parameters), there is also a need to construct the corresponding parametric circuit models, whose behaviour is characterized by the design and control parameters that describe the physical properties of the structure. The quality of these models, in terms of computation speed, accuracy and coverage of the design space, is key for the design, analysis and optimization of large-scale photonic circuits.

There are only a few publications in the literature that describe how to build accurate and efficient circuit models for passive components. The contributions^16,18,19^ demonstrate how to derive time-domain models for passive devices like waveguides and directional couplers; however, these models rely on simple analytic expressions that make it very difficult to capture complex photonic phenomena such as higher-order dispersion and wavelength-dependent effects. To address this issue, the techniques^17,20–22^ perform the time-domain modeling starting from the frequency-domain scattering parameters, therefore being capable of accurately modeling the aforementioned nonidealities. The convolution-based method^17^ directly applies inverse Fourier transform to the scattering parameters to obtain a non-parametric impulse response, which can be used for time-domain simulation. In practice though, the scattering parameters are band-limited or truncated, and this method often leads to violations of physical properties, such as causality and passivity^23^. The recently proposed work by Ye et al.^22^ on the other hand, performs the modeling by computing a stable and passive baseband state-space model via the robust *complex vector fitting* (CVF) algorithm. Such a model, which consists of a set of coupled ordinary differential equations (ODE) is supported by many commercial and open-source time-domain solvers. However, despite the attractive features of the latter technique, the models are non-parametric and their use is hence limited to performing circuit level design and optimization. For every design variation of the component, and even for each simulation at a different carrier wavelength, a new CVF model must be constructed, which often triggers one or more expensive EM simulation.

Thus, the literature exhibits a striking and practically relevant gap that this work seeks to fill. The novelty and the technical contribution of this work, which builds upon our previous research reported in Ye et al.^22^ and Ferranti et al.^24^, can be summarized as follows: (1) We derive a closed-form mathematical expression for the parameter dependency on the optical carrier frequency of the baseband macromodels^22^. This leads to the development of a wideband macromodel that can be simulated at any optical carrier frequency, making it suitable for the representation of multi-wavelength systems, (2) by leveraging the parameterization in terms of optical carrier frequency, the local interpolation scheme^25^, previously applied in microwave engineering, is extended with an additional frequency shifting coefficient for the computation of parametric baseband macromodels, capable of accurately predicting the complex behaviour of parametric passive photonic devices over the entire design space both in the frequency- and time-domain, (3) a wideband parametric baseband macromodel is proposed by combining the parameterization in terms of optical carrier frequency with design parameters. It is demonstrated that physical properties such as causality, stability, and passivity are preserved throughout the entire design space. Unlike traditional approaches that require numerous electromagnetic simulations with high computational costs for varying design parameters, our novel wideband parametric baseband model can be used to simulate device behavior as part of a circuit within a large continuous design space, eliminating the need for additional expensive EM simulations and significantly accelerating the design process. Notably, the presented parametric macromodel in this study can be seamlessly integrated into surrogate-based optimization frameworks^26^. To the author’s best knowledge, this is the first and only parametric macromodeling algorithm for photonic components reported in the literature to date.

## Baseband macromodeling

This section briefly summarizes the CVF algorithm^22^, which is one of the two major ingredients of the parametric macromodeling framework proposed in the paper. Although the concepts introduced here are not new, they are essential for understanding our work.

### Baseband equivalent signals and systems

Given the high frequencies of optical electromagnetic waves (200–300 THz), direct modelling of the EM waveform in the time domain is not a very efficient approach for circuit simulation, as it would require fs-scale time steps. Therefore, the common approach is to model the modulated envelope (in amplitude and phase) around a carrier frequency, which is sometimes referred to as a ‘baseband model’. The baseband modeling approach followed by the CVF algorithm^22^ allows one to adopt relatively large time steps when carrying out time-domain simulations, thereby significantly relaxing memory requirements and speeding up the computation time. In this section, the reader is introduced to the concept of baseband equivalent signals and systems since the theory is key for the derivation of the parametric model.

**Figure 1:**
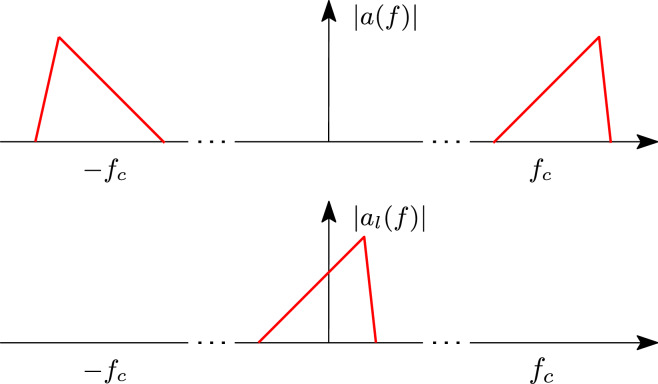
Example of the spectrum of a modulated optical signal (top) and its baseband equivalent representation (bottom).

The excitation signals of optical communication systems are usually defined as amplitude and/or phase modulated optical carriers in the form

1 $$\begin{aligned} a(t) = A(t) cos(2 \pi f_c t + \phi (t)) \end{aligned}$$

where $f_c$ is the optical carrier frequency and *A*(*t*) and $\phi (t)$ are the time-varying RF modulated amplitude and phase, respectively. In the frequency-domain, the spectrum of *a*(*t*) is centered around $f_c$ while its bandwidth is much smaller than $f_c$. Hence, signals in the form (1) are referred to as bandpass signals^27^. The baseband equivalent signal representation of (1) is

2 $$\begin{aligned} \begin{aligned} a_l(t)&= A(t) e^{j \phi (t)} \\ \end{aligned} \end{aligned}$$

which represents the complex envelope of the signal *a*(t) and it is widely used for the analysis of photonic systems. The signals *a*(*t*) and $a_l(t)$ are related by

3 $$\begin{aligned} \begin{aligned} a(t)&= {\mathfrak{R}} (a_l(t) e^{j 2 \pi f_c t}) \\ \end{aligned} \end{aligned}$$

where ${\mathfrak{R}} (\cdot )$ indicates the real part. The relation between *a*(*t*) and $a_l(t)$ in the frequency-domain is illustrated in Fig. 1.

**Figure 2:**
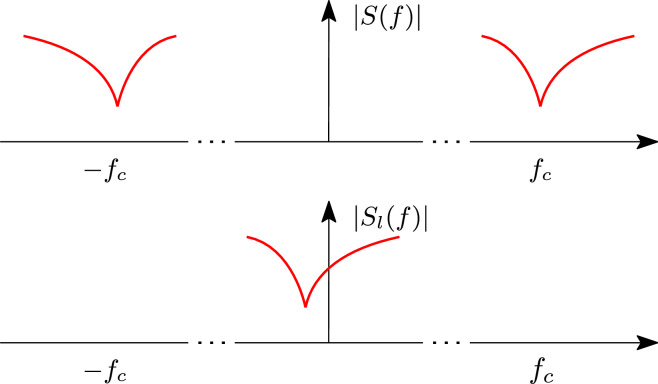
Example of the spectrum of a bandpass systems (top) and the corresponding baseband equivalent systems (bottom).

Now, the baseband equivalent of a bandpass system can be defined by applying the same concepts, as is illustrated in Fig. 2, where *S*(*f*) and $S_l(f)$ are the frequency response of the bandpass and baseband equivalent system respectively. Owing to the definition of bandpass signals and systems, and their baseband equivalents, it follows that the output of a bandpass system is intimately related to the output of the baseband equivalent system and the first can be analytically recovered from the latter.

### Complex vector fitting

The novel parametric modeling technique presented in this work relies on the CVF algorithm^22^, which starts from the scattering parameters of the photonic device under study in order to accurately capture non-ideal behaviours, such as higher-order dispersion, wavelength-dependent losses and backscattering. The scattering parameters are either acquired through measurements or can be simulated using electromagnetic modeling techniques such as finite difference time domain (FDTD)^3^, eigenmode expansion (EME)^4^, finite element (FE)^5^ or beam-propagation method (BPM)^6^. Once the scattering parameters have been obtained, their corresponding baseband representation is derived, and the CVF algorithm can be used to compute a model suitable for time-domain simulations, as described in the following.

Let us assume that the scattering parameters of a photonic device have been acquired by means of EM simulations for a discrete set of frequencies within the bandwidth of interest: ${\textbf{S}}(f_r)$ for $r = 1,\ldots , R$. The frequency response of the baseband equivalent system is then computed by shifting ${\textbf{S}}(f_r)$ to the imaginary axis by substituting $f_i = f_r - f_c$, where $f_c$ is the optical carrier frequency. The effect of this mathematical manipulation on the frequency response is illustrated in Fig. 2. Next, the baseband scattering parameters ${\textbf{S}}_l(f_i)$ are fed to the CVF algorithm, which builds a rational pole-residue model in the form^22^

4 $$\begin{aligned} {\textbf{S}}_l(s) = \sum _{k=0}^{K-1} \frac{\textbf{R}_{\textbf{k}}}{s-p_k} + {\textbf{D}} \end{aligned}$$

where $s = j 2 \pi f$ is the Laplace variable, ${\textbf{R}}_{\textbf{k}} \in {\mathbb {C}}^{n\times n}$ are the computed complex residues, $p_k$ are the complex poles, and ${\textbf{D}}\in {\mathbb {R}}^{n\times n}$ is a real matrix modeling the asymptotic response at high frequencies, where *n* is the number of ports of the system under study. Starting from the rational model (4), it is possible to analytically derive the corresponding system of *ordinary differential equations* (ODEs) in state-space form as^22^

5 $$\begin{aligned} {\left\{ \begin{array}{ll} \frac{\partial {\textbf{x}}_{\textbf{l}}({\textbf{t}})}{\partial t} = {\textbf{A x}}_{\textbf{l}}({\textbf{t}}) + {\textbf{B a}}_{\textbf{l}}({\textbf{t}}) \\ {\textbf{b}}_{\textbf{l}}({\textbf{t}}) = {\textbf{C x}}_{\textbf{l}}({\textbf{t}}) + {\textbf{D a}}_{\textbf{l}}({\textbf{t}}) \end{array}\right. } \end{aligned}$$

where ${\textbf{a}}_{\textbf{l}}({\textbf{t}}) \in {\mathbb {C}}^{n\times1}$ and ${\textbf{b}}_{\textbf{l}}({\textbf{t}}) \in {\mathbb {C}}^{n\times1}$ are the analytical forward and backward travelling waves of the *n*-port baseband system, corresponding to the RF modulated envelope of the photonic signal, ${\textbf{x}}_{\textbf{l}}({\textbf{t}}) \in {\mathbb {C}}^{m\times1}$ with $m=nK$ represents the state-variables, ${\textbf{A}} \in {\mathbb {C}}^{m\times m}$ is a diagonal matrix with $p_k$ at its non-zero entries, ${\textbf{B}} \in {\mathbb {C}}^{m\times n}$ is a matrix that only has zeros or ones, ${\textbf{C}} \in {\mathbb {C}}^{n \times m}$ is formed by horizontally stacking the residue matrices ${\textbf{R}}_{\textbf{k}}$ and ${\textbf{D}} \in {\mathbb {R}}^{n\times n}$ is the same matrix as in (4). The matrices ${\textbf{A}}, {\textbf{B}}, {\textbf{C}}$ and ${\textbf{D}}$ are called state-space matrices^22^. For future reference, it is worth noting that it is possible to express Eq. (4) in terms of the state-space matrices

6 $$\begin{aligned} {{\textbf{S}}_l(s)={\textbf{C}}\left( s {\textbf{I}}_m-{\textbf{A}} \right) ^{-1}{\textbf{B}}+ {\textbf{D}}} \end{aligned}$$

In this contribution, models in the form (4) and (5) are referred to as *macromodels* since they describe the behaviour of the system as seen from its input and output ports. Since the wideband parametric macromodeling technique, discussed in the subsequent sections, can be considered an extension of the non-parametric CVF technique explained here, we denote (5) as the reference CVF model.

#### Stability and passivity

When considering physical passive devices, like wavelength filters or other interferometric structures, it is essential for reliable time-domain simulation that the equivalent models are both stable and passive. If this is not the case, the mathematical representation can be the root cause of non-physical numerical instabilities during circuit simulations, especially when these circuits would contain feedback loops^28,29^. A complete discussion (including the derivation of) the conditions for stability and passivity of baseband rational models is given in Ye et al.^20,21^. In this section, we give an overview of the essential notions that will be used in the rest of the manuscript to evaluate stability and passivity of the proposed parametric modeling approach.

A state-space model is stable if the real part of all the eigenvalues of the ${\textbf{A}}$ matrix is negative. Since CVF builds pole-residue models having all complex poles with negative real part, stability is preserved by construction^22^.

In Ye et al.^20^, the passivity definition and conditions for complex-valued linear baseband systems are presented. In particular, there are two passivity constraints that the baseband scattering parameters $S_l(s)$ must satisfy:

1. ${\textbf{S}}_l(s)$ is analytic in ${\mathfrak{R}} (s) > 0$
2. ${\textbf{I}}_n - {\textbf{S}}_l^H(s) {\textbf{S}}_l(s)$ is a nonnegative matrix for all *s* such that ${\mathfrak{R}} (s) > 0$

An efficient and accurate method to assess the passivity of state-space models in the form (5) is based on the Hamiltonian matrix^20^ *M*, given by

7 $$\begin{aligned} {\textbf{M}} = \begin{bmatrix} {\textbf{M}}_{11} &{}\quad {\textbf{M}}_{12} \\ {\textbf{M}}_{21} &{}\quad {\textbf{M}}_{22} \end{bmatrix} \end{aligned}$$

where

8 $$\begin{aligned} \begin{array}{l} {\textbf{M}}_{11} = {\textbf{A}} - {\textbf{BL}}^{-1}{\textbf{D}}^H{\textbf{C}} \\ {\textbf{M}}_{12} = -{\textbf{BL}}^{-1}{\textbf{B}}^H \\ {\textbf{M}}_{21} = {\textbf{C}}^H{\textbf{Q}}^{-1}{\textbf{C}} \\ {\textbf{M}}_{22} = -{\textbf{A}}^H + {\textbf{C}}^H {\textbf{DL}}^{-1}{\textbf{B}}^H \\ {\textbf{L}} = {\textbf{D}}^H{\textbf{D}} - {\textbf{I}}_{n}, {\textbf{Q}} = {\textbf{DD}}^H- {\textbf{I}}_{n} \end{array} \end{aligned}$$

A state-space model is passive if its Hamiltonian matrix has no purely imaginary eigenvalues, since any imaginary eigenvalue indicates a crossover frequency where a singular value of the scattering matrix changes from being smaller to larger than unity, or vice versa^20,30^. After identification of the violating singular values by means of the Hamiltonian matrix, passivity can be enforced by perturbing the residues in such a manner that all singular values become smaller than unity^31^. It is useful to note that a publicly accessible Matlab implementation of the CVF algorithm^32^ is available at http://sumo.intec.ugent.be/CVF, including routines for passivity assessment and enforcement^31^ of the computed models. Finally, a stable and passive state-space model is causal by construction.

## Wideband baseband macromodeling

Prior to building the complex pole-residue model, the baseband scattering parameters $S_l(f_i)$ must be computed. This requires us to choose the value of the optical carrier frequency $f_c$, in order to shift the photonic frequency response to baseband. Hence, accurate time-domain simulations of CVF models are possible only if the optical carrier frequency of all excitation signals is equal to $f_c$. If a new value of the carrier frequency is chosen, a new model in the form (5) must be computed. Usually, it is not known upfront at which optical frequency(ies) the designers intend to perform simulations, so the modeling approach in Ye et al. is not very flexible and it does not scale well for systems with multiple wavelength channels. In order to overcome these limitations, in the following we define a novel CVF model parameterized with respect to the optical carrier frequency.

**Figure 3:**
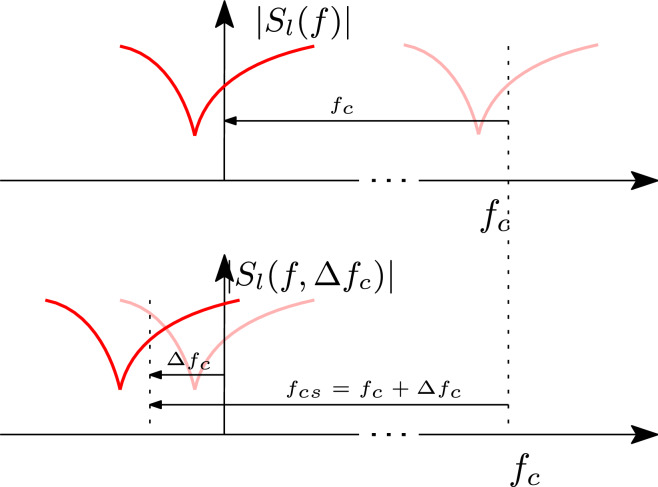
Example of spectrum of a baseband system (top) and its spectrum shifted by $\Delta f_c$ (bottom).

More specifically, starting from a CVF model computed for the carrier frequency $f_c$, our goal is to derive an analytic expression for a new set of state-space matrices that represent the baseband spectrum at carrier frequency $f_{cs} = f_c + \Delta f_c$, where $\Delta f_c$ is the desired frequency shift of the model that can be freely chosen by designers, as illustrated in Fig. 3. In this section, we demonstrate the derivation of the model and prove that both stability and passivity are preserved by construction.

While this is the first contribution describing a parametric CVF algorithm with respect to the optical carrier frequency, the idea of computing a rational model depending on $f_c$ is not new in the literature: the techniques in Ye et al.^20,21^ calculate a rational model at bandpass first, and then perform a frequency shift to obtain a corresponding baseband model. However, these approaches compute models of approximately double the size of the CVF algorithm for comparable accuracy^22^, and are more susceptible to numerical inaccuracies when performing time-domain simulations, since models obtained by these methods^20,21^ have non-zero frequency components centered around $-\,2f_c$. These limitations derive from the fact that the approaches in Ye et al.^20,21^ start from the bandpass scattering parameters and can not be applied directly to a baseband frequency representation.

### Model derivation

We start from the observation that multiplying the baseband signal $u_l$(t) with $e^{-j2 \pi \Delta f_c t}$ is equivalent to shifting the spectrum $U_l$(f) in the direction of negative frequencies by $\Delta f_c$

9 $$\begin{aligned} \begin{aligned} u_w(t) = u_l(t) e^{-j2 \pi \Delta f_c t} \end{aligned} \end{aligned}$$

**Figure 4:**
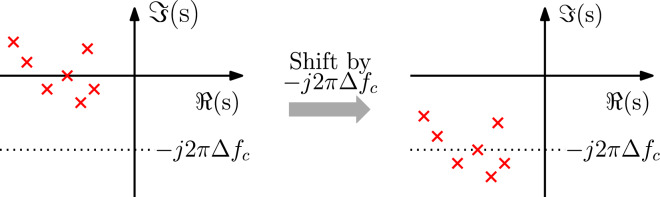
Example of poles of a model in form (5) representing the scattering parameters $S_l$(f) (left), and corresponding poles of the model (11) representing the scattering parameters $S_w$(f) (right).

Note that $u_w$(t) can be interpreted as the baseband equivalent signal of the bandpass signal *u*(*t*) at the optical carrier frequency $f_{cs} = f_c + \Delta f_c$. The next step is to express $a_l(t)$, $b_l(t)$ and $x_l(t)$ in the system of ODEs (5) in the form (9), which gives

10 $$\begin{aligned} \left\{ \begin{aligned} \frac{\text {d}{{{\textbf{x}}_{\textbf{w}}}(t)e^{j2 \pi \Delta f_c t}}}{\text {d}t}&= {{\textbf{Ax}}_{\textbf{w}}}(t)e^{j2 \pi \Delta f_c t}+{{\textbf{Ba}}_{\textbf{w}}}(t)e^{j2 \pi \Delta f_c t}\\ {{\textbf{b}}_{\textbf{w}}}(t)e^{j2 \pi \Delta f_c t}&= {{\textbf{C}}}{{\textbf{x}}_{\textbf{w}}}(t)e^{j2 \pi \Delta f_c t}+{{\textbf{Da}}_{\textbf{w}}}(t)e^{j2 \pi \Delta f_c t} \end{aligned} \right. \end{aligned}$$

where $a_w(t)$, $b_w(t)$ and $x_w(t)$ are the baseband equivalent signals defined according (9). By applying the chain rule and performing some simple mathematical manipulations, Eq. (10) can be formulated as

11 $$\begin{aligned} \left\{ \begin{aligned} \frac{\text {d}{{{\textbf{x}}_{\textbf{w}}}(t)}}{\text {d}t}&= ({{\textbf{A}}} - j2 \pi \Delta f_c {\textbf{I}}_m){{\textbf{x}}_{\textbf{w}}}(t)+{{\textbf{Ba}}_{\textbf{w}}}(t)\\ {{\textbf{b}}_{\textbf{w}}}(t)&= {{\textbf{Cx}}_{\textbf{w}}}(t)+{{\textbf{Da}}_{\textbf{w}}}(t) \end{aligned} \right. \end{aligned}$$

which represents a new baseband equivalent system at center frequency $f_{cs}$ by means of the state-space matrices (**A** - $j2\pi \Delta f_c {\textbf{I}}_m)$, **B**, **C** and **D**. Note that the macromodel (11), referred to as the *wideband baseband macromodel* in the rest of the manuscript, can be obtained by directly shifting all the poles of the state-space model (5) computed at the optical carrier frequency $f_c$ by $j2\pi \Delta f_c$ (see Fig. 4). Indeed, ${\textbf {A}}$ is a diagonal matrix, with the poles in (4) as diagonal elements. It is important to note that, by tuning the parameter $\Delta f_c$, it is possible to vary the baseband carrier frequency $f_{cs} = f_c + \Delta f_c$ of the model (11) as needed. The transfer function of the wideband baseband macromodel is given by

12 $$\begin{aligned} \hat{{\textbf{S}}}_l(f, \Delta f_c)={\textbf{C}}\left( j2\pi (f + \Delta f_c){\textbf{I}}_m-{\textbf{A}} \right) ^{-1}{\textbf{B}}+ {\textbf{D}}={\textbf{S}}(f + f_{cs}) \end{aligned}$$

### Stability and passivity

As previously stated, a state-space model is stable if the real part of all the eigenvalues of the ${\textbf {A}}$ matrix is negative. Let us define $\tilde{{\textbf {A}}}={\textbf {A}} - j2\pi \Delta f_c {\textbf{I}}_m$. Note that the frequency shift in $\tilde{{\textbf {A}}}$ does not modify the real part of the eigenvalues of ${\textbf {A}}$. Hence, if the reference CVF model (5) is stable, the wideband baseband macromodel (11) is stable as well, by construction.

The passivity of the wideband baseband macromodel can be verified by computing the eigenvalues of its Hamiltonian matrix. The Hamiltonian matrix $M_w$ of the wideband baseband macromodel can be obtained by substituting the state-space matrices in (11) into Eqs. (7) and (8). Subsequently, by performing some simple mathematical manipulations, we can express ${\textbf{M}}_w$ in terms of the Hamiltonian matrix ${\textbf{M}}$ corresponding to the model (5) as

13 $$\begin{aligned} {\textbf{M}}_w = {\textbf{M}} - j2\pi \Delta f_c {\textbf{I}}_{2m} \end{aligned}$$

Now, assuming that the matrix ${\textbf{M}}$ has eigenvalues $\lambda _{i}$, it must hold that

14 $$\begin{aligned} \lambda _{i} \begin{bmatrix} x_{1} \\ x_{2} \end{bmatrix} = {\textbf{M}} \begin{bmatrix} x_{1} \\ x_{2} \end{bmatrix} = \lambda _{wi} \begin{bmatrix} x_{1} \\ x_{2} \end{bmatrix} +j2\pi \Delta f_c \begin{bmatrix} x_{1} \\ x_{2} \end{bmatrix} \end{aligned}$$

which indicates that

15 $$\begin{aligned} \begin{array}{ll} \lambda _{wi} = \lambda _{i} - j2\pi \Delta f_c,&\quad \text {for }i=1,\ldots ,2m \end{array} \end{aligned}$$

Hence, the wideband baseband macromodel (11) is passive, i.e. the Hamiltonian matrix ${\textbf{M}}_w$ has no purely imaginary eigenvalues, if the reference state-space model (5) is passive, i.e. the Hamiltonian matrix ${\textbf{M}}$ has no purely imaginary eigenvalues.

### Wideband macromodeling strategy

**Figure 5:**
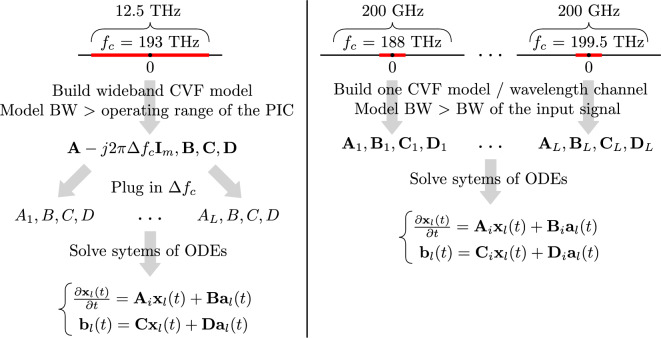
Example of the wideband baseband macromodeling framework (left) and the reference CVF^22^ (right). The bandwidth is indicated with the acronym BW.

In order to take advantage of the modeling flexibility offered by the novel wideband approach, it may be useful to consider a large range of frequencies when computing the model, for example the entire operating range of the PIC under study, typically defined between 187.5 and 200 THz. From a mathematical standpoint, there are no restrictions on the values of $\Delta f_c$ as the baseband macromodel is well-defined across all frequencies. However, in practice, to ensure the accuracy of time-domain simulations, it is essential to satisfy $f_{min} + BW_{sig} / 2 \ge f_c + \Delta f_c \ge f_{max} - BW_{sig} / 2$, where $f_{min}$ and $f_{max}$ define the range of optical frequencies for which the macromodel is computed, and $BW_{sig}$ is the bandwidth corresponding to the spectrum of the modulated optical input signals ${\textbf{a}}_{\textbf{w}}(t)$ of the state-space model (11). In this framework, given the aforementioned condition is respected, a designer can freely choose the value of the carrier frequency to be used for time-domain simulations without having to compute a new state-space model. This is a difference with respect to the reference CVF^22^: since a new rational model must be computed for every value of interest of the carrier frequency, it is beneficial to consider a more limited frequency range, corresponding only to the spectrum of the modulated optical input signals around the carrier frequency (normally a few hundred gigahertz). Indeed, considering a smaller frequency range may reduce the number of poles (and thus the size of the the corresponding system of ODEs) needed by the model to reach the desired accuracy in the entire bandwidth. Hence, for a similar accuracy, a wideband baseband macromodel can offer higher flexibility compared to the reference CVF approach, but it can have a larger computational complexity. Both modeling methodologies are illustrated in Fig. 5.

## Parametric baseband macromodeling

### Parametric macromodeling

Parametric macromodels accurately predict the complex behaviour of high-speed multiport systems that are characterized by frequency and other design variables^33^. Unfortunately, the calculation of such models is not a trivial task. In particular, guaranteeing model passivity uniformly over the entire design space introduces substantial technical challenges and makes it difficult to build parametric macromodels that are concurrently accurate, compact and passive.

The literature describes two principal strategies: *root macromodels interpolation*^24,25,34^ and *multivariate rational fitting*^33,35–37^. The first strategy constructs a guaranteed passive parametric macromodel by interpolating a set of passive frequency-dependent macromodels (called root macromodels), computed for different parameter configurations in the design space. Despite this approach being robust and straightforward, it is not very compact and the number of poles of the resulting model is generally higher than the number of poles of each of the macromodels. The second approach aims to compute an implicit and global parameterization of the complex dynamic behaviour by means of multivariate rational fitting. Main advantages of this technique are the compact formulation of the parametric numerator and denominator in a closed form analytic expression and the model order that does not scale with the dimension of the design space. The main drawback with many of the proposed multivariate fitting schemes is the passivity enforcement, which either may miss small passivity violations or is known to be over-conservative, leading to a model with reduced accuracy^37^.

In the literature, the aforementioned parametric macromodeling techniques have all been presented in the context of *vector fitting* (VF)^38^, a technique extensively used for the modeling of distributed microwave devices. Since the VF technique operates at bandpass, it is *not applicable* for the modeling and simulations of PICs. To overcome this limitation, we propose to build parametric baseband macromodels via the CVF modeling procedure^22^, based on root macromodels interpolation.

### Parametric baseband rational models

The proposed modeling technique is a baseband adaptation of Ferranti et al.^24^ and relies on the computation of several frequency-dependent rational pole residue models for different parameter sets in the design space. Whereas in Ferranti et al.^24^ the rational models are computed by means of VF, given that we are targeting circuits operating at optical frequencies, the models here are built following the CVF algorithm^22^. The result of this initial step is a set of univariate macromodels, stable and passive, which we call root macromodels, and which serve as starting point to build the desired parametric macromodel.

**Figure 6:**
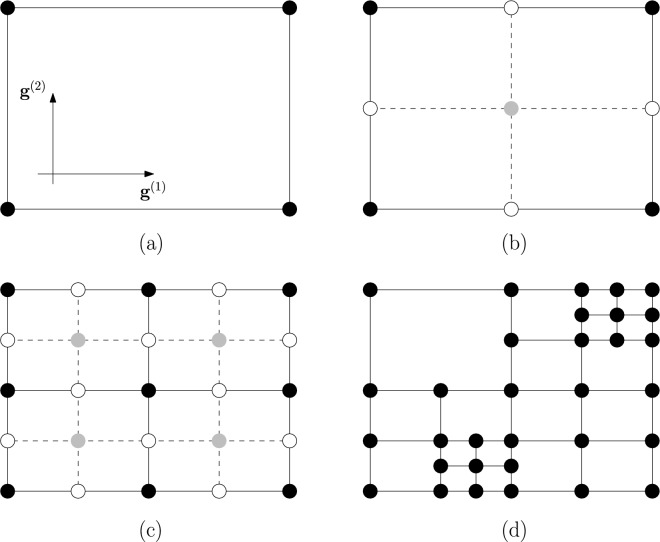
Example of sequential sampling in parameters $g^{(1)}$ and $g^{(2)}$. The black points represent the computed root macromodels at each stage of the iterative procedure. The gray points in the design space are used to verify the accuracy of the root macromodels interpolation, whereas the white ones are the points where new root macromodels will be computed. (**a**) Represents the initial sampling grid; (**b**,**c**) Show the evaluation of the parametric baseband macromodel at the center of the different subregions in the first and second iteration respectively; (**d**) illustrates the final sampling grid.

Note that the choice of the points in the design space where to compute the root macromodels can be automated by following a *sequential sampling* approach^39,40^. The main idea is to partition the design space in suitable sub-regions, for example hyperrectangles. Only the vertexes of the hyperrectangles are chosen to compute root macromodels in pole/residue form. Then, in each point within the chosen hyperrectangle a model in pole/residue form can be calculated by interpolating the root macromodels that define its vertexes. These macromodels can then be used for frequency- or time-domain simulations. This choice makes the technique in Ferranti et al.^24^ a local interpolation scheme: only few macromodels determine the model’s predictions in a small portion of the overall design space. Note that, in contrast, in global interpolation approaches all the macromodels computed in the entire design space contribute to the model’s predictions. If the accuracy of the macromodel, defined in this work as the maximum absolute error (MAE) between the original frequency response ${\textbf{S}}_l$(s,**g**) and the estimated response by the parametric baseband macromodel ${\textbf{R}}_l$(s,**g**), is not sufficient in a specific sub-region, that portion of the design space is divided in smaller hyperrectangles, and the modeling procedure is iterated again until the desired accuracy is reached in the entire design space. The main advantage of this modeling strategy is that a designer has only to choose the desired accuracy and the parametric macromodel is built in an automated way. The sequential sampling scheme used in this work is described in details in Chemmangat et al.^39^. An application example of this modeling strategy in a design space of two variables is given in Fig. 6.

Let us denote an *M*-dimensional cell-region of the design space as $\Omega _i$, and the corresponding vertices as ${\textbf{g}}_k^{\Omega _i}, k=1,\ldots ,M^2$. Then, in order to improve the prediction accuracy of the interpolated model, for each vertex root macromodel ${\textbf{R}}_l\left( s, {\textbf{g}}_k^{\Omega _i}\right)$ a set of amplitude scaling $\alpha _k({\textbf{g}}_j^{\Omega _i}),j=1\ldots ,M^2$ and frequency scaling $\beta _k({\textbf{g}}_j^{\Omega _i}),j=1\ldots ,M^2$ real-valued coefficients are computed that make the root macromodel an accurate approximant of the other root macromodels within that cell. Subsequently, a multilinear interpolation function is used to parameterize the coefficients in the design space

16 $$\begin{aligned} {\textbf{T}}\left( {\textbf{g}}\right) = \sum _{k=1}^{M^2} T({\textbf{g}}_k^{\Omega _i}) l_k^{\Omega _i}({\textbf{g}}) \end{aligned}$$

with ${\textbf{T}}\left( {\textbf{g}}\right)$ representing the interpolated coefficient and $l_k^{\Omega _i}({\textbf{g}})$ the piecewise linear interpolation kernel. The multivariate representation in Ferranti et al.^24^ at a point **g** is then obtained as a combination of root macromodels and corresponding amplitude and frequency scaling coefficients by adopting the same multilinear interpolation scheme used to parameterize the scaling coefficients

17 $$\begin{aligned} {\textbf{R}}_l\left( s, {\textbf{g}}\right) = \sum _{k=1}^{M^2} \alpha _k ({\textbf{g}}){\textbf{R}}_l\left( s\beta _k({\textbf{g}}), {\textbf{g}}_k^{\Omega _i}\right) l_k^{\Omega _i}({\textbf{g}}) \end{aligned}$$

The amplitude and frequency scaling coefficients introduced in Ferranti et al.^24^ perform well at bandpass for the modeling of microwave devices, but as we will explain, their modeling capabilities are significantly reduced when applied at the baseband for the representation of PICs. Photonic scattering parameters $S\left( j(\omega _c + \Delta \omega )\right)$ are typically defined in a narrow frequency band around the center frequency, i.e. $\Vert \Delta \omega \Vert \ll \omega _c$. The compressed or expanded photonic scattering response $S\left( \beta (j\omega _c + \Delta \omega )\right)$ is obtained by multiplying the Laplace variable *s* with the frequency scaling coefficient $\beta$. Now, suppose $\beta = 1 + b$ with $\Vert b \Vert \ll 1$, then

18 $$\begin{aligned} \begin{aligned} S\left( \beta j(\omega _c + \Delta \omega )\right)&= S\left( j\omega _c + j\Delta \omega + j b \omega _c + j b \Delta \omega )\right) \\&\approx S\left( j(\omega - \Delta \omega _c) \right) \end{aligned} \end{aligned}$$

with $\Delta \omega _c = b \omega _c$. Note that the second equation of (18) is obtained by neglecting the term $b \Delta \omega$. The above result implies that a scaled passband model, computed at terahertz frequencies, is capable of modeling large frequency shifts, but will perform poorly when used to represent PICs whose frequency response exhibits a large compression or expansion over the design space. Applying the same frequency transformation to the baseband CVF models modifies the scattering response $S_l$ in a fundamental different way: While subject to a large compression or expansion, the scattering response $S_l$ will show almost no frequency shift because $\omega _c \approx 0$. Now, since the scattering parameters of passive PICs often contain a resonance behaviour that is controlled by one or more design parameters, the latter observation will substantially limit the modeling capabilities of the parametric baseband macromodel. To this end, we introduce the additional frequency shifting coefficient $\gamma _k({\textbf{g}})$ and propose a parametric baseband macromodel of the form

19 $$\begin{aligned} {\textbf{R}}_l\left( s, {\textbf{g}}\right) = \sum _{k=1}^{M^2} \alpha _k ({\textbf{g}}){\textbf{R}}_l\left( s\beta _k({\textbf{g}}) - j \gamma _k({\textbf{g}}), {\textbf{g}}_k^{\Omega _i}\right) l_k^{\Omega _i}({\textbf{g}}) \end{aligned}$$

The set of amplitude scaling $\alpha _k({\textbf{g}}_j^{\Omega _i}),j=1\ldots ,M^2$, frequency scaling $\beta _k({\textbf{g}}_j^{\Omega _i}),j=1\ldots ,M^2$ and frequency shifting $\gamma _k({\textbf{g}}_j^{\Omega _i}),j=1\ldots ,M^2$ coefficients required by the multilinear interpolation scheme (16) for the parameterization of $\alpha _k({\textbf{g}})$, $\beta _k({\textbf{g}})$ and $\gamma _k({\textbf{g}})$ respectively, are found by means of optimization, such that

20 $$\begin{aligned} \alpha _k({\textbf{g}}_j^{\Omega _i}) {\textbf{R}}_l\left( \beta _k({\textbf{g}}_j^{\Omega _i})s - j \gamma _k({\textbf{g}}_j^{\Omega _i}), {\textbf{g}}_k^{\Omega _i}\right) = {\textbf{R}}_l\left( s, {\textbf{g}}_j^{\Omega _i}\right) ,\quad j\ne k \end{aligned}$$

Our experimental findings suggest to limit the range for $\gamma _k({\textbf{g}}_j^{\Omega _i})$ to $[-\gamma _0, \gamma _0]$ and set the frequency range for evaluation of the parametric macromodel during the sequential sampling as $[f_{min} + \gamma _0, f_{max} - \gamma _0]$. Here, $f_{min}$ and $f_{max}$ represent the optical frequency range for which the root macromodels are computed. This strategy ensures high accuracy of the frequency-shifted root macromodels across the entire bandwidth of the parametric macromodel, i.e. $[f_{min} + \gamma _0, f_{max} - \gamma _0]$. Note that if $j=k$, it follows that $\alpha _k({\textbf{g}}_j^{\Omega _i}) = \beta _k({\textbf{g}}_j^{\Omega _i}) = 1$ and $\gamma _k({\textbf{g}}_j^{\Omega _i}) = 0$. Now, since the parametric baseband macromodel (19) can be considered as a linear combination of amplitude and frequency transformed state-space representations, it is possible to express the model (19) in state-space form.

21 $$\begin{aligned} {\textbf{R}}_l\left( s, {\textbf{g}}\right) = {\textbf{C}}\left( {\textbf{g}}\right) \left( s {\textbf{I}} - {\textbf{A}}\left( {\textbf{g}}\right) \right) ^{-1}{\textbf{B}}\left( {\textbf{g}}\right) + {\textbf{D}}\left( {\textbf{g}}\right) \end{aligned}$$

with

22 $$\begin{aligned} \begin{array}{ll} {\textbf {A}}\left( {\textbf{g}}\right) = \begin{bmatrix} \beta _1({\textbf{g}})^{-1}({\textbf{A}}_1^{\Omega _i} - j \gamma _1({\textbf{g}})) &{}\quad \hdots &{}\quad {\textbf{0}}\\ \vdots &{}\quad \ddots &{}\quad \vdots \\ {\textbf{0}} &{}\quad \hdots &{}\quad \beta _{M^2}({\textbf{g}})^{-1}\left( {\textbf{A}}_{M^2}^{\Omega _i} - j \gamma _{M^2}({\textbf{g}})\right) \\ \end{bmatrix}, &{}\quad {\textbf{B}}\left( {\textbf{g}}\right) = \begin{bmatrix} {\textbf{B}}^{\Omega _i}_1 \\ \vdots \\ {\textbf{B}}^{\Omega _i}_{M^2} \end{bmatrix}, \\ {\textbf{C}}\left( {\textbf{g}}\right) = \begin{bmatrix} \alpha _1({\textbf{g}})\beta _1({\textbf{g}})^{-1} {\textbf{l}}^{\Omega _i}_{1}({\textbf{g}}) {\textbf{C}}^{\Omega _i}_1 &{}\quad \hdots &{}\quad \alpha _{M^2}({\textbf{g}})\beta _{M^2}({\textbf{g}})^{-1}{\textbf{l}}^{\Omega _i}_{M^2}({\textbf{g}}) {\textbf{C}}^{\Omega _i}_{M^2} \end{bmatrix}, \\ {\textbf{D}}\left( {\textbf{g}}\right) = \alpha _1({\textbf{g}}) {\textbf{l}}^{\Omega _i}_{1}({\textbf{g}}) {\textbf{D}}^{\Omega _i}_1 +\cdots + \alpha _{M^2}({\textbf{g}}) {\textbf{l}}^{\Omega _i}_{M^2}({\textbf{g}}) {\textbf{D}}^{\Omega _i}_{M^2} \end{array} \end{aligned}$$

The main advantage of the state-space form (21) is that it can be directly adopted for time-domain simulation. It should be noted though that the number of root macromodels used by the local interpolation scheme scales quadratically with the dimension of the design space *M*. This means that, for devices with a dynamic and wideband frequency response that require root macromodels with a high number of poles, the state-space model (22) can accumulate a significant number of states in scenarios where the design space dimension is high. This, in turn, results in a large system of ODEs, leading to computationally expensive time-domain simulations and imposing restrictions on the applicability of the proposed methodology. Another challenge in higher dimensions is the generation of extensive chunks of EM data, which incurs significant computational costs. The curse of dimensionality is a pervasive issue and an ongoing challenge in the domain of parametric macromodeling techniques.

### Stability and passivity preserving interpolation of the transformation coefficients

The amplitude and frequency scaled coefficients in Ferranti et al.^24^ are shown to preserve stability and passivity over the entire design space. However, these rational models represent bandpass scattering parameters with *physical meaning*: such scattering parameters have Hermitian symmetry with respect to positive and negative frequencies and the corresponding impulse response is real-valued. Because passivity conditions are defined differently for real-valued linear bandpass systems than for their complex-valued baseband equivalents^22^, which are the study of this work, it must be verified that a passive baseband system remains passive if an amplitude scaling coefficient $\alpha$, frequency scaling coefficient $\beta$ and frequency shifting coefficient $\gamma$ are applied to it.

Following a similar approach as the one adopted in Ferranti et al.^24^ it is verified that the passivity conditions, as outlined earlier in this paper, are preserved when the root macromodels are modified with an amplitude and frequency scaling. It is easy to understand that if $\beta ({\textbf{g}}_k^{\Omega _i}) \ge 0$ and $\alpha ({\textbf{g}}_k^{\Omega _i}) \ge 0$, the first passivity condition is preserved. Since the multilinear interpolation kernel belongs to a special class of positive interpolators, it satisfies the following constraints

23 $$\begin{aligned} \begin{aligned} l_{k}^{\Omega _i}({\textbf{g}})&\ge 0 \\ l_{k}^{\Omega _i}\left( {\textbf{g}}_j^{\Omega _i}\right)&= \delta _{k,j} \\ \sum _{k=1}^{M^2}l_k^{\Omega _i}({\textbf{g}})&= 1 \end{aligned} \end{aligned}$$

The second passivity condition, which ensures no energy is generated by the system, is equivalent to the condition $\Vert S_l(s) \Vert _{\infty } \le 1$ ($H_{\infty }$ norm), that is, the largest singular value of $S_l(s)$ does not exceed one in the right-half complex plane. Now, since $\alpha _k \left( {\textbf{g}}\right)$ is parameterized according (16), we can write

24 $$\begin{aligned} \begin{aligned} \alpha _k \left( {\textbf{g}}\right)&= \sum _{j=1}^{M^2} \alpha _k({\textbf{g}}_j^{\Omega _i}) l_j^{\Omega _i}({\textbf{g}}) \end{aligned} \end{aligned}$$

Then, by imposing that $\alpha _k({\textbf{g}}_j^{\Omega _i}) \le 1 / \Vert R_l(s,{\textbf{g}}_k^{\Omega _i}) \Vert _{\infty }, j=1..M^2$ and using the properties of (23), it is possible to define an upper bound on $\alpha _k \left( {\textbf{g}}\right)$

25 $$\begin{aligned} \begin{aligned} \alpha _k \left( {\textbf{g}}\right)&\le 1 / \Vert R_l(s,{\textbf{g}}_k^{\Omega _i}) \Vert _{\infty } \sum _{j=1}^{M^2} l_j^{\Omega _i}({\textbf{g}}) \\&\le 1 / \Vert R_l(s,{\textbf{g}}_k^{\Omega _i}) \Vert _{\infty } \end{aligned} \end{aligned}$$

which we can use to show that

26 $$\begin{aligned} \Vert \alpha _k\left( {\textbf{g}}\right) R_l(s\beta _k \left( {\textbf{g}}\right) ) \Vert _{\infty } \le \alpha _k \left( {\textbf{g}}\right) \Vert R_l(s\beta _k \left( {\textbf{g}}\right) ) \Vert _{\infty } \le 1 \end{aligned}$$

Therefore it follows that passivity is preserved if $\alpha _k({\textbf{g}}_j^{\Omega _i})$ and $\beta _k({\textbf{g}}_j^{\Omega _i})$ satisfy

27a $$\begin{aligned} 0 \le \alpha ({\textbf{g}}_k^{\Omega _i})&\le 1 / \Vert R_l(s,{\textbf{g}}_k^{\Omega _i}) \Vert _{\infty } \end{aligned}$$

27b $$\begin{aligned} \beta ({\textbf{g}}_k^{\Omega _i})&\ge 0 \end{aligned}$$

It is important to note that in Ferranti et al.^24^, $\alpha$ must be smaller than unity for passivity to hold, which is a stricter constraint than the condition proposed here. The amplitude coefficient is mainly introduced for the modeling of frequency responses that preserve their shape, but show different attenuation/gain over the design space. However, by restricting $\alpha$ to be smaller than one, a vertex root macromodel can only be accurately transformed into another vertex root macromodel along the direction of attenuation ($\alpha \le 1$). To overcome this limitation and to improve the modeling capability of the technique, the relaxed constrained (27a) is introduced. Since the infinity norm can be efficiently computed in scripting languages such Matlab or Python, the relaxed constraint will have minimal affect on the computational runtime.

The parameter $\gamma$ is introduced in this paper for a more accurate and compact modeling of photonic scattering representations and it is responsible for a shift of the frequency response with respect to the Laplace variable *s*. When this transformation is applied to the amplitude and frequency scaled macromodel $\alpha _k\left( {\textbf{g}}\right) R_l(s\beta _k \left( {\textbf{g}}\right) )$, it results in a frequency shift of the singular values without affecting the infinity norm of the macromodel. As a consequence, it must follow that $\Vert \alpha _k\left( {\textbf{g}}\right) R_l(s\beta _k \left( {\textbf{g}}\right) - j \gamma _k \left( {\textbf{g}}\right) ) \Vert _{\infty } = \Vert \alpha _k\left( {\textbf{g}}\right) R_l(s\beta _k \left( {\textbf{g}}\right) ) \Vert _{\infty } \le 1$. Thus, we can conclude that the modified CVF model, subject to amplitude, frequency scaling and frequency shifting transformations, is guaranteed to be passive over the entire design space.

### Stability and passivity preserving interpolation of root macromodels

Concerning the multilinear interpolation of the root macromodels, the first passivity condition is always satisfied since it is imposed on the modified root macromodels used in the interpolation. Now, since the modified root macromodels are guaranteed to be passive over the entire design space, indicating that there infinity norm is less than one, i.e. $\left\| \alpha _k ({\textbf{g}}){\textbf{R}}_l\left( s\beta _k({\textbf{g}}) - j\gamma _k({\textbf{g}}), {\textbf{g}}_k^{\Omega _i}\right) \right\| _{\infty } \le 1$, and by using the properties of the piecewise multilinear interpolation scheme, see (23), we can write

28 $$\begin{aligned} \begin{aligned} \left\| {\textbf{R}}_l\left( s, {\textbf{g}}\right) \right\| _{\infty }&\le \sum _{k=1}^{M^2} \left\| \alpha _k ({\textbf{g}}){\textbf{R}}_l\left( s\beta _k({\textbf{g}}) - j \gamma _k({\textbf{g}}), {\textbf{g}}_k^{\Omega _i}\right) \right\| _{\infty } l_k^{\Omega _i}({\textbf{g}}) \\&\le \sum _{k=1}^{M^2} l_k^{\Omega _i}({\textbf{g}}) \\&\le 1 \end{aligned} \end{aligned}$$

thereby proving that the parametric baseband macromodel (19) is passive.

## Wideband parametric baseband macromodeling

The proposed wideband parametric baseband macromodeling framework relies on the interpolation of a set of optimized root macromodels for the representation of photonic scattering parameters that depend on design variables (such as layout and substrate features). Following the sequential sampling strategy^39,40^, the scattering parameters of the PIC are evaluated in the frequency range of interest for different points of a hyperrectangular grid in the design space. A fixed value of the optical carrier frequency $f_c$, is used to shift the data samples to the baseband. Given the set of scattered data samples $\{(s,{\textbf{g}})_k, {\textbf{S}}_l(s, {\textbf{g}})_k\}_{k=1}^{K_{tot}}$, the root macromodels are computed via CVF and their coefficients are refined through an optimization routine. Passivity of the root macromodels is checked, and, eventually enforced as a post-processing step^31^. Finally, a multilinear interpolation scheme is adopted to parameterize the transformation coefficients and integrate the optimized root macromodels, leading to the construction of the parametric baseband macromodel (19).

The resulting parametric baseband macromodel can be used by standard optimization routines for fast and precise evaluation of the scattering response in the design space. Once a good parameter configuration is identified, the parametric baseband macromodel is evaluated and converted into the state-space representation (22), yielding a system of ODEs that can be solved in a wide range of simulators. Additionally, if we want to perform time-domain simulation at the optical carrier frequency $f_{cs} = f_c + \Delta f_c$, the poles at the diagonal entries of the matrix ${\textbf {A}}$ (22), must be shifted by $j2\pi \Delta f_{c}$. The state-space representation (22) gives rise to a system of complex-valued ODEs, which can only be simulated in solvers that support complex-valued signals and matrices. However, Ye et al.^22^ demonstrated that the complex-valued model (5) can be analytically transformed into a real-valued model, making it compatible with a broader range of simulators, such as Verilog-A. The same conversion method can be applied to the state-space representations presented in this work (22). Moreover, it’s important to note that SPICE-based solvers do not directly accept differential equations as input. For a detailed discussion on how to convert a CVF macromodel into an equivalent electrical network and how to simulate this equivalent circuit within a SPICE environment, the reader is referred to the author’s previous work^41^. Note that we refer to macromodels that combine the wideband macromodeling approach with the parametric macromodeling technique as *wideband parametric baseband macromodels*.

## Numerical results

This section presents two application examples of the proposed modeling and simulation techniques. The scattering parameters of the photonic circuits under study are evaluated via the Caphe circuit simulator (Luceda Photonics) and electromagnetic simulations in Lumerical FDTD Solutions (Ansys).

### 4-Channel arrayed waveguide grating

**Figure 7:**
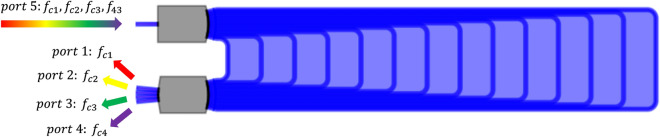
AWG: Layout and operation principle of the 4-channel AWG demultiplexing filter.

**Figure 8:**
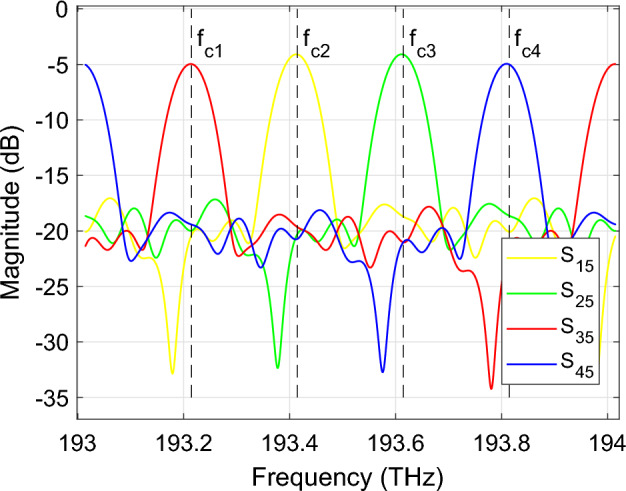
AWG: Transmission spectrum of the 4-channel AWG demultiplexing filter.

In this numerical example we discuss the time-domain simulation of a 4-channel arrayed waveguide grating (AWG), used to separate four RF signals modulated on different optical carriers. The Layout of the AWG, comprising the input star coupler, the output star coupler and a bundle of waveguides, is designed and simulated using the AWG Designer module from Luceda IPKISS. The star couplers of the AWG are simulated by computing the field output of the input apertures with CAMFR, Luceda’s eigenmode expansion tool, propagating it in the free propagation region, and performing mode overlaps to collect the fields at the output apertures. The delay lines are modeled as linear dispersive waveguides whereas dispersion in the star couplers is ignored and they are only simulated for a single wavelength. The final layout and transmission spectrum of the AWG is illustrated in Figs. 7 and 8 respectively. The primary source of crosstalk in the AWG is imperfect imaging due to phase errors that arise from sidewall roughness: when the phases in the waveguides are not perfect, the image will be distorted and light gets coupled to other output waveguides. In order to properly simulate this type of behaviour, the waveguide delay lines are modeled with phase errors corresponding to a stochastic linewidth variation of 2 nm in the iSiPP50G silicon photonics platform. The time-domain cross-talk between the different channels is simulated using both the wideband baseband macromodel (11) and the reference CVF model (5) to verify the validity of the proposed modeling technique.

**Figure 9:**
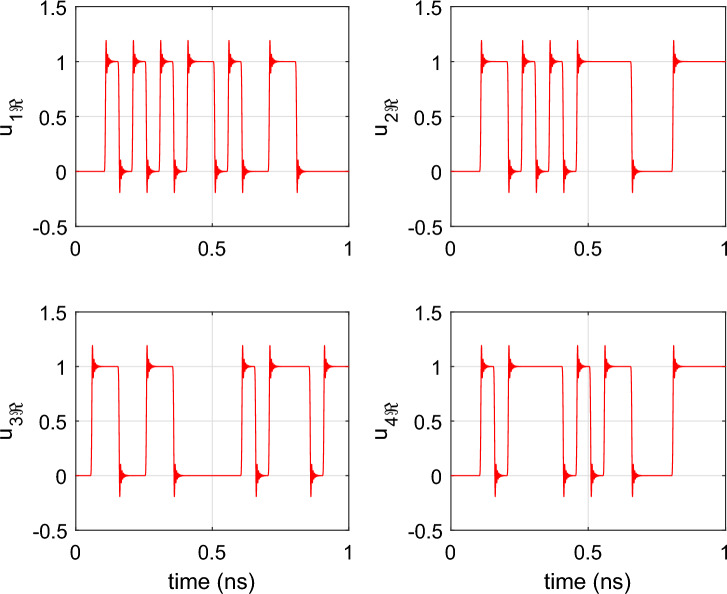
AWG: The OOK excitation signals at port 5 used to perform time-domain simulation of the 4-channel AWG.

The in-phase component of the input port (P5) is excited with four on-off keying (OOK) signals $u_i(t), i=1,..,4$ with optical carrier frequencies $f_{ci} = {193.01}\,{\textrm{THz}} + i \cdot {200}$ GHz, corresponding to the center wavelengths of the AWG channels. The 20 Gbs and 20 bit long PRBS signals are simulated in Keysight ADS and are passed to a Butterworth low-pass filter to have a 3-dB bandwidth of 400 GHz, matching the channel spacing of the filter. Note that this configuration closely matches the behavior of real-world communication systems, in which the modulator and driver electronics restrict the frequency range of the optical signals. The excitation signals are illustrated in Fig. 9. By opting for the OOK modulation scheme, the signal detection is incoherent which allows to simulate the cross-talk by simply adding up the signal power leaking from the different channels.

The scattering parameters of the device are evaluated in Luceda IPKISS over the frequency range [193.01; 194.01] THz. In this example, 1001 frequency samples are used, and they are uniformly distributed over the frequency range of interest. Next, the scattering parameters are shifted to baseband using $f_c = {194.51}\,{\textrm{THz}}$. Following the CVF modeling procedure, a stable and passive CVF model is built with 24 poles, leading to a maximum modeling error between the data and the model response below ${-\,61}$ dB. Then, after conversion of the rational representation in the state-space model (5), the diagonal entries of the complex-valued ${\textbf{A}}$ matrix are shifted by $-j 2 \pi \Delta f_c$ to obtain the wideband baseband macromodel (11), which can be used to perform time-domain simulation at any arbitrary optical carrier frequency in the range [193.01 + $BW_{mod} / 2$; 194.01 − $BW_{mod} / 2$]  THz, where $BW_{mod}$ is the modulation bandwidth. Considering the bandwidth of the OOK input signals is about 400 GHz, this means the wideband baseband macromodel can be used in the frequency range [193.21 ; 193.81]. In particular, the wideband baseband macromodel is evaluated for $\Delta f_{c} = {-\,500}\,{\textrm{GHz}} + i \cdot {200}\,{\textrm{GHz}}$, yielding four state-space models that represent the AWG at its different wavelength channels.

Next to the wideband baseband macromodel, which is valid over a broad frequency range, four reference CVF models with 14 poles and a BW of ${400}\,{\textrm{GHz}}$ are computed for each wavelength channel of the AWG. Note that this is done for comparison reasons. Because the reference CVF models have a smaller bandwidth than the wideband baseband macromodel, they require less poles to achieve the same accuracy, resulting in a smaller system of ODEs that is more efficient to simulate.

**Figure 10:**
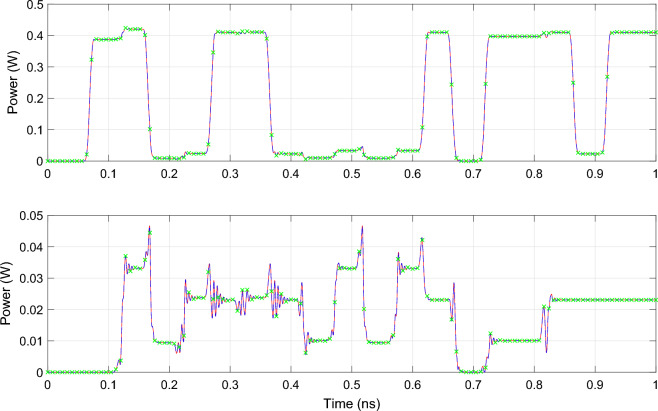
AWG: The total signal power (top) and cross-talk (bottom) detected at port 3 of the AWG. The red line represents the simulation results obtained in Matlab with the reference CVF model, the blue dashed line and the green markers represent the simulation results obtained in Matlab and Caphe respectively, with the wideband CVF model.

**Figure 11:**
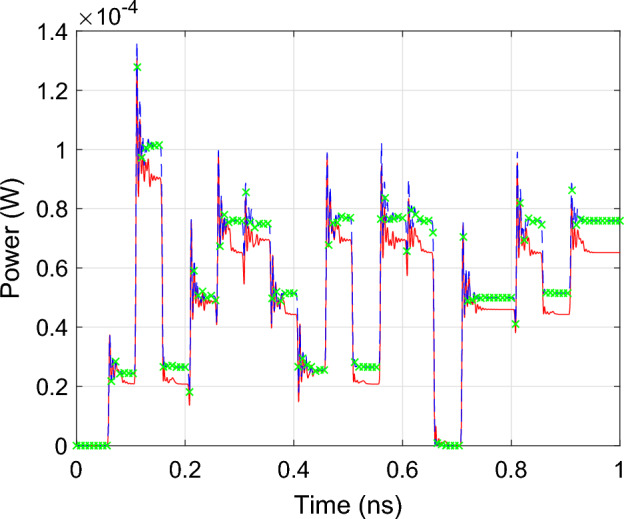
AWG: The reflected power at the input port (P5) of the AWG. The red line represents the simulation results obtained in Matlab with the reference CVF model, the blue dashed line and the green markers represent the simulation results obtained in Matlab and Caphe respectively, with the wideband CVF model.

Once all CVF models are computed, transient simulation of the 4-channel AWG is performed by solving the systems of ODEs in Matlab using the *lsim* routine. To demonstrate the compatibility of the proposed technique with commercial photonic circuit simulators, the wideband baseband model (11) is also simulated in Caphe, the circuit simulator of Luceda photonics. All time-domain simulations are executed on a personal computer with Intel Core i7 processor and 16 GB RAM. The simulated total signal power and cross-talk at port 3 of the AWG are illustrated in Fig. 10. Note that the cross-talk at port 3 is identified as the signal power emanating from channels 1, 2 and 4 distorting the demultiplexed signal transmitted over channel 3. Because the bandwidth of the electronic excitation signals is matched to the channel spacing of the filter, i.e. 400 GHz, the observed cross-talk is limited and it will still be possible to decode the detected bit sequence. The reflected signal power observed at the input port (P5) of the AWG is illustrated in Fig. 11. Taking a closer look at Fig. 11, it can be observed that the deviation between the wideband baseband macromodel and the reference CVF model is of the order $10^{-5}$ while the signal is only of the order $10^{-4}$. This significant deviation is the result of the modeling accuracy in the frequency domain, which was set to about ${-\,60}$ dB and is relatively low for an accurate modeling of the $S_{55}$ parameter with an average value of about $-\,45$ dB. Table 1 summarizes the run time and accuracy of the different simulations. The accuracy of the wideband baseband macromodel, defined as the maximum absolute error with respect to the reference CVF model, is less than $8.0e{-}4$ in Matlab and less than $5.8e{-}3$ in Caphe, hence the conclusion that simulation of the wideband baseband macromodel converges very well and yields accurate results in both environments. One challenge in embedding a state-space model with matrices *A*, *B*, *C* and *D* in a Caphe circuit model is that using a regular ODE solver for these types of matrices is not the most efficient. The *lsim* routine in MATLAB adopts the state propagation method, which has some ‘tricks’ to work with these matrices and is a much more efficient technique in terms of computational effort than regular ODE solvers (related to taking the exponent of a matrix, $exp(A*dt)$). Because of this reason, simulation of the state-space model in Caphe is less efficient and takes about ten times longer compared to solving the model with *lsim*.

**Table 1:** AWG: Performance metrics of the wideband CVF and CVF model of the 4-channel AWG.

Models	Simulation time (s)	Accuracy
Reference CVF (Matlab)	0.13	–
Wideband CVF (Matlab)	0.21	8.0e−4
Wideband CVF (Caphe)	3.24	5.8e−3

### Double ring resonator

**Figure 12:**
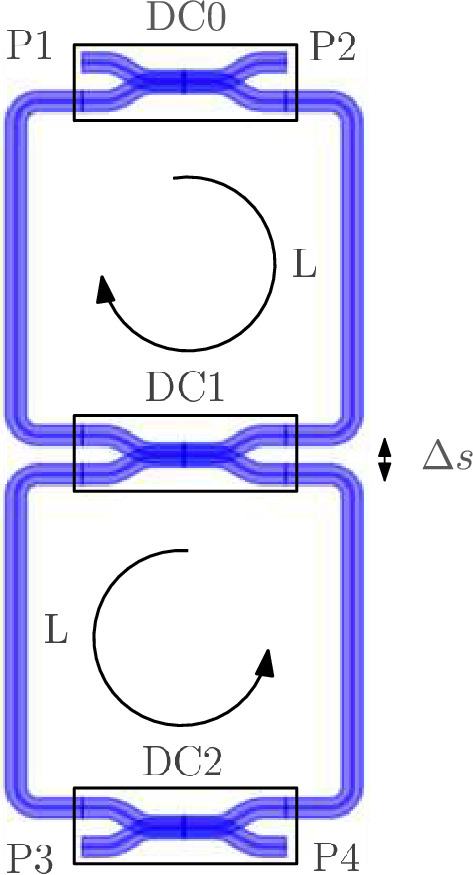
DRR: Layout of the SOI double ring resonator.

The wideband parametric baseband macromodeling technique is used to model the scattering parameters of a ${220}\,{\hbox {nm}}$ silicon-on-insulator (SOI) double ring resonator, comprising three directional couplers (DCs) connected by strip waveguides. The layout of the filter is illustrated in Fig. 12. A wideband parametric baseband macromodel is computed as a function of the varying gap between the waveguides of DC1 $\Delta s \in [{166}{}, {262}{}]\, {}{\hbox {nm}}$ and the varying roundtrip length of the ring resonators $L \in [{313.3}{}, {313.6}{}]\, {}{\upmu \hbox {m}}$. The gap between the waveguides of the outer directional couplers is kept fixed at ${550}\,{\textrm{nm}}$ over the frequency range [193.2, 193.6]  THz.

The scattering parameters of the DCs are simulated in Lumerical FDTD. To obtain reliable simulation results, a uniform mesh with mesh sizes of 50 nm, 3 nm and 50 nm in x, y and z directions, respectively, is selected. It should be noted that the mesh size determines both the accuracy and the computational cost of the FDTD simulations. Convergence tests however showed that the aforementioned configuration strikes a good trade-off between accuracy and computational efficiency. The waveguides on the other hand are modeled by their effective index $n_{eff}$ = 2.27, group index $n_g$ = 4.54 and losses $\alpha$ = 1.91 dB/cm. Using the Luceda design software, the scattering parameters of each component are then combined into a single overall S-matrix that describes propagation through the double ring resonator.

A consequence of the low-loss structure, whose scattering parameters have an infinity norm very close to one, and small errors in the FDTD simulations, due to effects such as staircasing and grid dispersion^42^, is that the simulated scattering parameters show small-passivity violations. Though the Matlab toolbox implements routines for passivity enforcement, both the rate at which these algorithms converge and the size of the error resulting from the correction, depend on the degree of the passivity violations. Hence, to ease the computation of passive and accurate root macromodels, the passivity of the tabulated data is restored by truncating the violating singular values^43^.

The wideband parametric baseband macromodel is computed as proposed earlier in this work. The baseband frequency shift is arbitrarily chosen ($f_c = {193.41}\,{\textrm{THz}}$). A standard bottom-up approach is used to select the required number of poles. In order to achieve a model that is both compact and accurate, the error threshold, adopted by the sequential sampling algorithm, is set to − 40 dB. Running the proposed algorithm in the aforementioned configuration, a wideband parametric baseband macromodel is built that consists of 74 root macromodels. Since the algorithm only considers the error at the center of the hyperrectangular subspaces, its effective accuracy, evaluated over a dense uniform $17 \times 17$ grid, is slightly lower than the predefined − 40 dB, i.e. − 38.7 dB. It must be noted that the CPU time needed for the execution of the algorithm is small compared to the CPU time needed to generate the tabulated data. This is mainly due to the FDTD simulations which are very costly in terms of computation.

To demonstrate the necessity of the frequency shift coefficient in the wideband parametric baseband modeling framework, a second model is computed where the root macromodels are only modified by an amplitude and frequency scaling coefficient, i.e. a direct baseband adaptation of the scalable models in Chemmangat et al.^39^. The results obtained with both models are summarized in Table 2. While model 2, comprising a similar number of root macromodels as model 1, successfully manages to represent the dependency of the scattering parameters on $\Delta s$, for reasons highlighted earlier in this paper, it fails to represent the varying center frequency of the filter controlled by the roundtrip length *L*, resulting in a poor accuracy.

**Table 2:** DRR: Comparison of the wideband parametric baseband model proposed in this work (Model 1) with the direct baseband adaptation of the scalable models^39^ (Model 2).

Technique	Wideband parametric baseband macromodeling	Baseband scalable macromodeling^44^
Frequency shift	Yes	No
Number of root macromodels	71	72
S-parameter evaluations	123	127
FDTD simulations (DC)	17	17
Accuracy (MAE) (dB)	− 38.67 dB	− 2.65
CPU time (s)		
Data generation	303,497	303,508
Algorithm execution	1774	781

**Figure 13:**
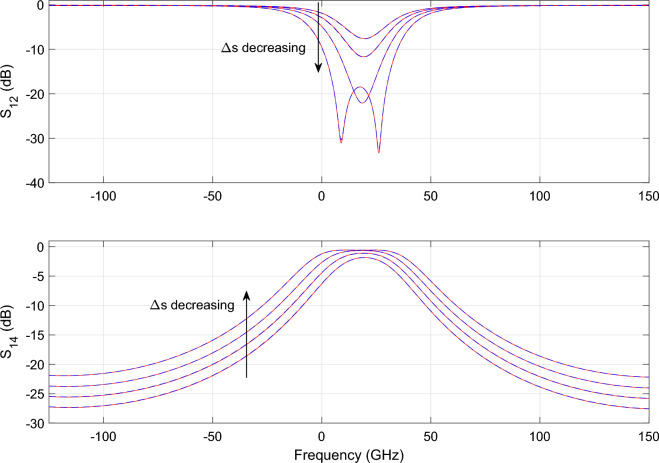
DRR: Magnitude of the $S_{12}$ (top) and $S_{14}$ (bottom) scattering parameters as a function of $\Delta s$ while L is fixed at ${313.43}\,{\upmu \hbox {m}}$. The blue line represents the simulated response while the red dashed line represents the response generated with the wideband parametric baseband macromodel.

**Figure 14:**
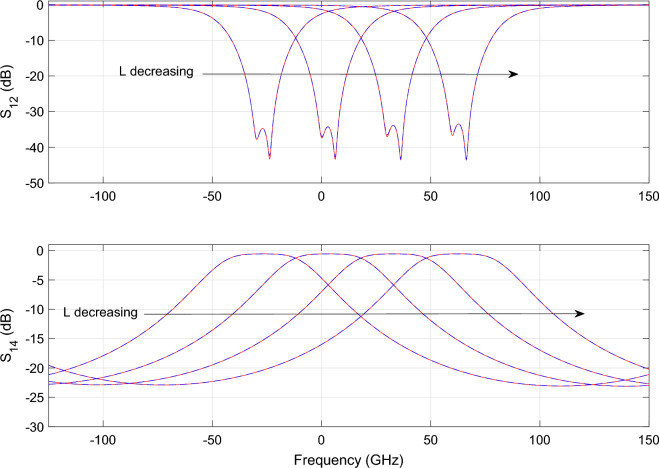
DRR: Magnitude of the $S_{12}$ (top) and $S_{14}$ (bottom) scattering parameters as a function of L while $\Delta s$ is fixed at ${634}\,{\hbox {nm}}$. The blue line represents the simulated response while the red dashed line represents the response generated with the wideband parametric baseband macromodel.

**Figure 15:**
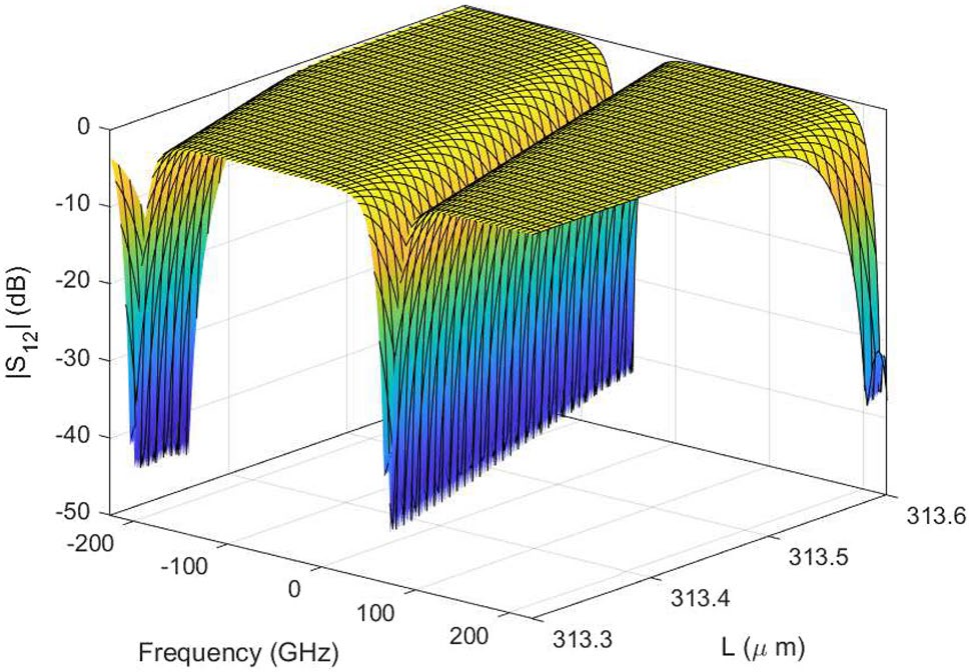
DRR: Magnitude plot of the $S_{12}$ scattering parameter as a function of $\Delta s$ for L = ${313.43}\,{\upmu \hbox {m}}$ generated with the wideband parametric baseband macromodel.

**Figure 16:**
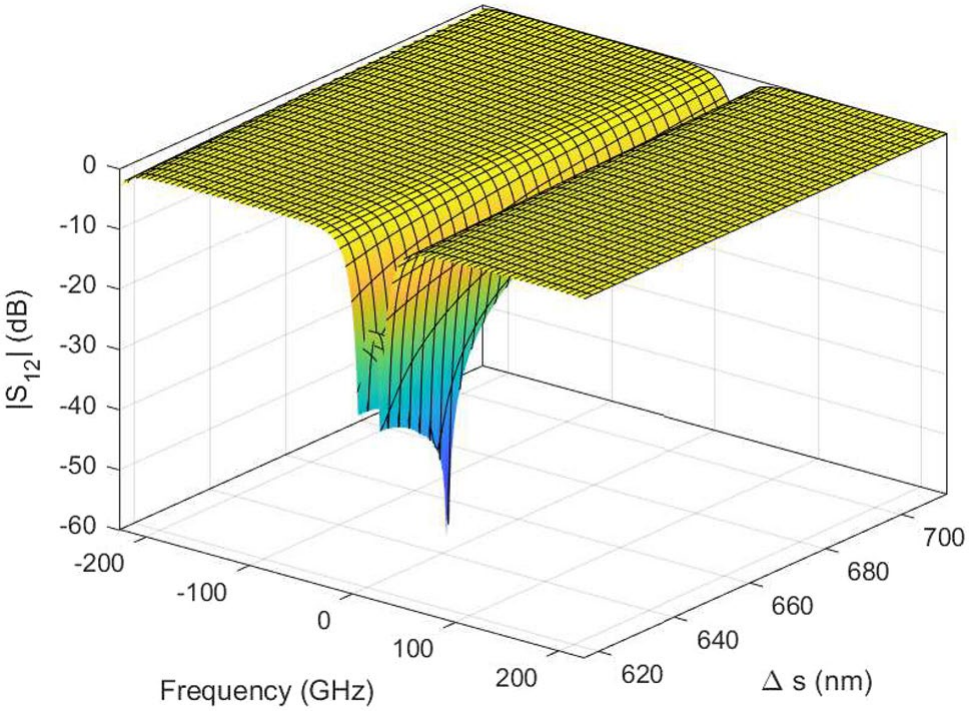
DRR: Magnitude plot the $S_{12}$ scattering parameter as a function of L while for $\Delta s$ = ${634}\,{\hbox {nm}}$ generated with the wideband parametric baseband macromodel.

**Figure 17:**
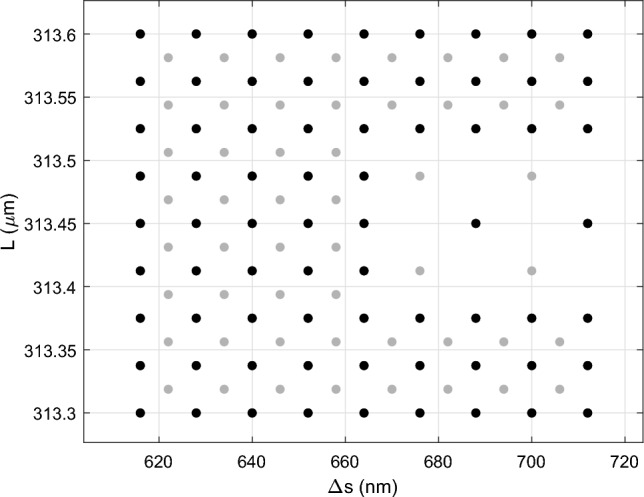
DRR: Design space sampling; the black dots represent the root macromodels; the gray dots represent the parameter combinations for which the wideband parametric baseband model has been evaluated by means of EM simulation.

Figure 13 shows the parametric behavior of the magnitude of $S_{12}$ and $S_{14}$ as a function of $\Delta s$ and frequency for L = ${313.43}\,{\upmu \hbox {m}}$. Similarly, Fig. 14 shows the magnitude of $S_{12}$ and $S_{14}$ as a function of *L* and frequency for ${634}\,{\hbox {nm}}$. Figures 15 and 16 show the surface plots for Figs. 13 and 14, respectively. The sampling in the design space is illustrated in Fig. 17.

**Figure 18:**
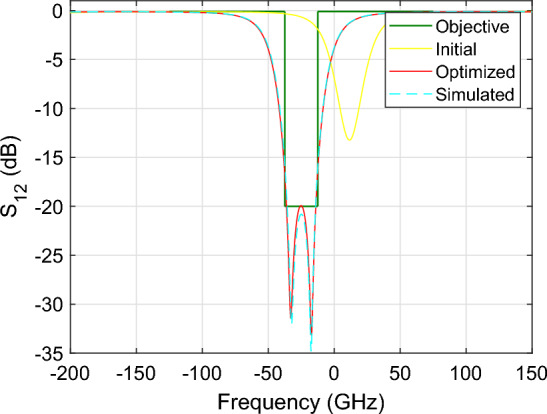
DRR: Magnitude of the $S_{12}$ scattering parameter before and after the optimization.

Now that we have computed the wideband parametric baseband macromodel, we can use it to optimize the spectral response of the photonic filter. The specifications for the bandpass filter are are expressed in terms of the scattering parameter $S_{12}$

29 $$\begin{aligned} \begin{array}{ll} |S_{12} |< -\,20\,\textrm{dB} &{}\quad \text {for } f_0 - 12.5\, \textrm{GHz}< f< f_0 + 12.5 \, \textrm{GHz} \\ |S_{12} |> -\,0.1\,\textrm{dB} &{}\quad \text {for } f_0 - 100 \, \textrm{GHz}< f< f_0 - 12.5 \, \textrm{GHz} \\ |S_{12} |> -\,0.1\,\textrm{dB} &{}\quad \text {for } f_0 + 12.5 \, \textrm{GHz}< f < f_0 + 100 \, \textrm{GHz} \end{array} \end{aligned}$$

with $f_0 = {193.39}\,{\textrm{THz}}$ the center frequency of the filter. Translating the requirements (29) to baseband, they are combined into a single objective function $g(\Delta s, L, f)$

30 $$\begin{aligned} \begin{array}{ll} g(\Delta s, L, f) = 10 \max {(20log_{10}(|S_{12} |), -20)} &{}\quad \text {for } f_{l0} - 12.5 \, \textrm{GHz}< f< f_{l0} + 12.5 \, \textrm{GHz} \\ g(\Delta s, L, f) = \max {(20log_{10}(|S_{12} |), -\,0.1)} &{}\quad \text {for } f_{l0} - 100 \, \textrm{GHz}< f< f_{l0} - 12.5 \, \textrm{GHz} \\ g(\Delta s, L, f) = \max {(20log_{10}(|S_{12} |), -\,0.1)} &{}\quad \text {for } f_{l0} + 12.5 \, \textrm{GHz}< f < f_{l0} + 100 \, \textrm{GHz} \\ \end{array} \end{aligned}$$

with $f_{l0} = f_0 - f_c$. The objective function (30) is subsequently defined and minimized by means of the Matlab *fmincon* method. The optimization routine was able to finish in ${5.6}\,{s}$ and performed 61 function evaluations to find that the parameter configuration resulting in the lowest objective value is $\Delta s$ = ${622}\,{\hbox {nm}}$ and $L = {313.537}\,{\upmu \hbox {m}}$. By utilizing a wideband parametric baseband macromodel as a substitute for costly EM simulations, the evaluation of the objective function is done very efficiently, resulting in a significant reduction of CPU time required for the optimization process. It is estimated that using a direct interface with the EM simulator would have resulted in a total CPU time of 302 h. It should be noted that this number might seem large compared to the 85 h required to build the parametric macromodel, as reported in Table 2. The reason is that the adaptive sampling algorithm adopted by the parametric macromodeling technique, takes samples on a rectangular grid, see Fig. 17. Now, since the scattering response of the entire double ring resonator is calculated by combining the frequency response of its building blocks, i.e. the directional couplers and waveguides, only 17 FDTD simulations, instead of 123, were necessary to build the parametric macromodel. However, the samples taken by the genetic optimizer in Matlab will lie scattered in the design space, and as a consequence one FDTD simulation is needed for each function evaluation, i.e. 67 in total, resulting in the reported computational run time of 302 h. The magnitude of the initial and optimized scattering parameter $S_{12}$ is illustrated in Fig. 18. It is important to emphasize that the delta between the optimized response, computed via EM simulations, and the response generated with the wideband parametric baseband macromodel, is not a limitation of the proposed modeling technique but the result of numerical dispersion in the FDTD simulations.

**Figure 19:**
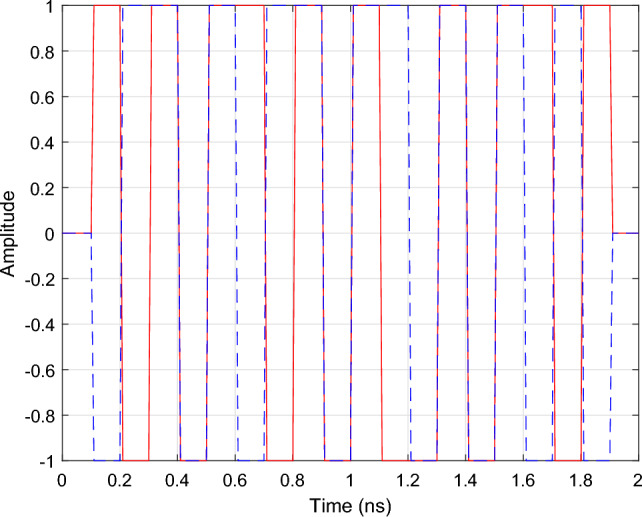
DRR: The in-phase part (red) and quadrature part (blue) of the 10 Gbit/s and 12 bits long 4-QAM input signal applied to P1 of the DRR.

**Figure 20:**
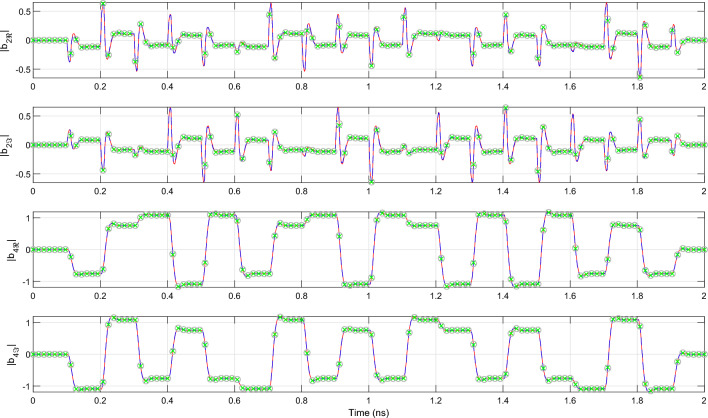
DRR: The transient output response at the pass (P2) and drop (P4) port of the double ring resonator filter. The red lines represent the simulation results of the reference CVF model while the blue dashed lines represent that of the wideband parametric baseband macromodel obtain using the Matlab *lsim* routine. The grey and green markers represent the simulation results obtained in Caphe and SPICE respectively.

To demonstrate that the wideband parametric baseband macromodel is suited for the time-domain characterization of PICs, a transient simulation of the optimized filter response is performed. Setting $\Delta s$ and *L* equal to their optimized values, the parametric model is converted into a state-space model of the form (22) with 50 poles. Because it is desired to characterize the filter at the center of its pass band, the diagonal entries of the ${\textbf{A}}$ matrix are shifted by $-j 2 \pi (f_0 - f_c)$. A transient simulation is set up by exciting the input port *P*1 of the filter with the ${10}\,{\mathrm{Gbaud/s}}$ 4-QAM signal depicted in Fig. 19. To verify the validity of (22), a compact state-space model (5) is built via the reference CVF technique. The reference CVF model, characterized by 11 poles and trained on the optimized scattering response computed via EM simulation, achieves an accuracy of − 49.6 dB. Figure 20 shows the transient output response of the in-phase and quadrature signals at the pass and drop port of the double ring filter obtained with the two models. The wideband parametric baseband macromodel (22) shows good convergence with the reference CVF model, and the overall error, averaged over time, is equal to 9.4e−3. Again note that the primary source for this error is not the accuracy of the wideband parametric baseband macromodel, but the numerical dispersion in the FDTD simulator. Additionally, the computational time required to simulate the model (22) is 0.6 s, while the reference CVF model only requires 0.06 s. It should be noted that the CPU time required to solve a system of ODEs directly scales with the number of poles, which, by construction, will always be higher for the parametric baseband macromodel. To demonstrate the compatibility of the proposed modeling framework with various circuit simulators, the optimized macromodel is also simulated in Caphe and SPICE. For conversion of the CVF model to a SPICE-compatible netlist, we refer the reader to our previous work^41^. Note that the poles of the state-space model must be shifted before building the equivalent electrical netlist. The computational time required to simulate the model (22) in Caphe and SPICE is 2.41 s and 4.85 s respectively. The simulation in SPICE is less efficient, as it is a modified nodal solver and falls into the category of regular ODE solvers^41^. As already highlighted in the previous example, the same can be said about Caphe’s ODE solver. While the SPICE and Caphe solvers are a bit slower, these environments offer the possibility to connect CVF models with active devices such as modulators and amplifiers, enabling designers to simulate dispersive circuits with a mix of passive/active components. Though this hasn’t been investigated yet, this will be the topic of future research.

## Conclusion

This paper presented a wideband parametric baseband macromodeling technique for the representation of linear and passive photonic devices whose scattering parameters depend on design variables such as geometrical layout or substrate features. The analytic parameterization with respect to the optical carrier frequency allows the adoption of the model for the simulation of multi-wavelength systems. The proposed technique has several advantages over existing methods, including its ability to capture complex photonic phenomena such as dispersion, backscattering and wavelength-dependent effects, while preserving physical properties such as stability and passivity over the entire design space. Additionally, the baseband model can be converted into a state-space representation for efficient time-domain characterization. An alternative approach to the one proposed in this study involves breaking down a device into a collection of building blocks (such as directional couplers, waveguides, phase shifters, etc.) and developing a parametric macromodel for each of these individual components. This would enable users to construct more extensive and intricate circuits using these pre-evaluated primitives, potentially offering a more scalable solution, especially within the context of the foundry model. However, this strategy demands further investigation and will be explored as a topic for future research.

### Supplementary Information

Supplementary Information.

## Data Availability

All data generated or analysed during this study are included in this published article (and its Supplementary File)
